# Health care professionals' knowledge and attitudes toward telemedicine

**DOI:** 10.3389/fpubh.2023.957681

**Published:** 2023-02-16

**Authors:** Muhammad Salman Bashir, Diana Selvamony Lalithabai, Shahad AlOtaiby, Amani Abu-Shaheen

**Affiliations:** ^1^Department of Biostatistics, Research Services Administration Research Center, King Fahad Medical City, Riyadh, Saudi Arabia; ^2^Nursing Education and Practice Improvement Administration, King Fahad Medical City, Riyadh, Saudi Arabia; ^3^Department of Scientific Writing and Publication, Research Services Administration Research Center, King Fahad Medical City, Riyadh, Saudi Arabia

**Keywords:** E-health, telemedicine, knowledge, attitude, health care professionals

## Abstract

**Background:**

The utilization of modern communication technology in the healthcare field is known as telemedicine, and it represents an advancement in the healthcare industry. For effective implementation of these technologies, healthcare professionals must possess the appropriate knowledge and hold a positive perspective toward the implementation of telemedicine. The current study aims to evaluate the knowledge and perspective of healthcare professionals in King Fahad Medical City, Saudi Arabia toward telemedicine.

**Methods:**

This study was carried out in a diverse hospital, King Fahad Medical City, Saudi Arabia and it was a cross-sectional study. The study took place from June 2019 until February 2020, during which 370 healthcare professionals, including physicians, nurses, and other healthcare professionals participated. The data was gathered by using a structured self-administered questionnaire.

**Results:**

The analysis of the data revealed that the majority of the healthcare professionals who participated in the study, 237 (63.7%), had limited knowledge of telemedicine. About 41 (11%) participants had a good understanding of the technology, and 94 participants (25.3%) had extensive knowledge. The overall attitude of the participants toward telemedicine was positive, with a mean score of 3.26. The mean attitude scores varied significantly (*P* < 0.001) among the different professions, with physicians scoring 3.69, allied healthcare professionals scoring 3.31, and nurses scoring 3.07. The coefficient of determination (R2) was used to evaluate the variation in attitude toward telemedicine and it was found that education (12.4%) and nationality (4.7%) had the least impact on the attitude toward telemedicine.

**Conclusion:**

Healthcare professionals are crucial to the successful implementation and continuity of telemedicine. However, despite their positive attitude toward telemedicine, most of the healthcare professionals who participated in the study had limited knowledge of it. There were differences in attitude among different groups of healthcare professionals. As a result, it is necessary to create specialized educational programs for healthcare professionals to guarantee the proper implementation and continuation of telemedicine.

## 1. Introduction

Telemedicine is a technology that enables the delivery of healthcare and the exchange of healthcare information over long distances. As technology and the world evolve, telemedicine has become more prevalent, particularly due to advances in telecommunications and healthcare. Despite high demand for telemedicine services, there are still challenges that need to be addressed such as clinician availability and service delivery. Additionally, factors such as internet availability, security, and workflow can have an impact on telemedicine. Despite these challenges, it is expected that telemedicine will have a positive impact on healthcare, similar to how the personal computer revolutionized office work. Telemedicine can improve access to healthcare, healthcare standards and the efficiency of healthcare delivery with only a little improvement.

Telemedicine is a powerful technology that uses telecommunication and information systems to deliver health care services. It is widely accepted across the globe because of its extensive applications and benefits. At present, the utilization of E-health applications has significantly increased throughout the healthcare delivery system of many countries (1). Several programs have been introduced in developed countries and reported rapidly for 10–15 years e.g., UK, Finland, Europe, Taiwan, and worldwide. Some recent programs are also dedicated to medical education, such as Western Australia. The healthcare industry and medical organizations worldwide have predominantly engaged in utilizing telemedicine technology to improve and expand the existing medical services and patient care system. Thus, the contemporary approach is to improve the efficiency of telemedicine applications in terms of speed, ease to use, and affordability (2). Currently the traditional health care system strives to enhance the efficiency, equity, and contribution to cost-effectiveness in healthcare, which can potentially be achieved through the implementation of specific telemedicine applications, such as remote patient monitoring and remote healthcare delivery (3).

The usage of telemedicine took a surge after the outbreak of COVID-19 pandemic when both patients and healthcare professionals were figuring out ways to access and deliver medical services safely (4). The increased development of digital technologies further boosted the importance of implementing telemedicine. At the present time there are numerous issues in the healthcare system of Saudi Arabia including a shortage of healthcare professionals, limited financial resources, and an increased demand for healthcare services. Moreover, the changing pattern of disease from infectious to non-infectious pathologies and poor access to healthcare services has casted an additional burden on the health care system in Saudi Arabia (5). The main health problems of the country include non-communicable diseases like diabetes, hypertension, cardiovascular diseases, and obesity (6). Furthermore, there are future challenges that must be overcome, such as ensuring a sustainable financial system and workforce planning issues, including the provision of high-quality training and the realignment of health services to adapt and respond to changing demographics and disease patterns (7). Thus, telemedicine is a solution to curb most of the present and future challenges facing the healthcare system of Saudi Arabia by offering new solutions for rural healthcare services and bridge the disparity in quality and accessibility of health care between urban and rural regions (8). Recently, the Ministry of Health Saudi Arabia has launched a project to build a centralized national electronic health record database and create a national electronic medical system (9). The Kingdom of Saudi Arabia is also marching toward establishing a research center for E-health service implementation (10). These projects clearly illustrate the potential of telemedicine in the medical industry and its ability to improve the overall healthcare system of Saudi Arabia. The current large government health care system in Saudi Arabia has an extensive geographical coverage and aims to provide E-health services to target population. It is also significant to provide free healthcare services for individuals, families, and communities in remote areas. Therefore, the implementation of telemedicine technology in such settings will offer many benefits, including the ability to cater the needs of communities in remote areas and provide improved health care services to larger population (11).

The success of a new technology, such as telemedicine, depends on various factors including the understanding and skills of professionals, their attitudes, and the work environment. In order for telemedicine to be adopted, it is crucial to train professionals and assess their readiness to provide these services. Telemedicine is a new technology in the healthcare sector, and understanding the knowledge and attitudes of healthcare professionals toward it is important for facilitating its adoption. The researcher aims to evaluate the knowledge and attitudes of healthcare professionals toward telemedicine. Healthcare professionals play a vital role in the success of telemedicine by ensuring proper implementation of information technology, facilitating a smooth transition and encouraging the use of this technology in the future. Given the need for telemedicine services in Saudi Arabia, this study aims to examine the knowledge and attitudes of healthcare professionals toward telemedicine and assess their readiness to provide such services. The goal of this study is to aid in the successful implementation of telemedicine technology and ensure its long-term viability in the medical industry. As the field of e-health services and telemedicine continues to grow globally, the findings of this study will contribute to both theoretical knowledge and practical applications.

## 2. Materials and methods

### 2.1. Study design

**Figure 1:**
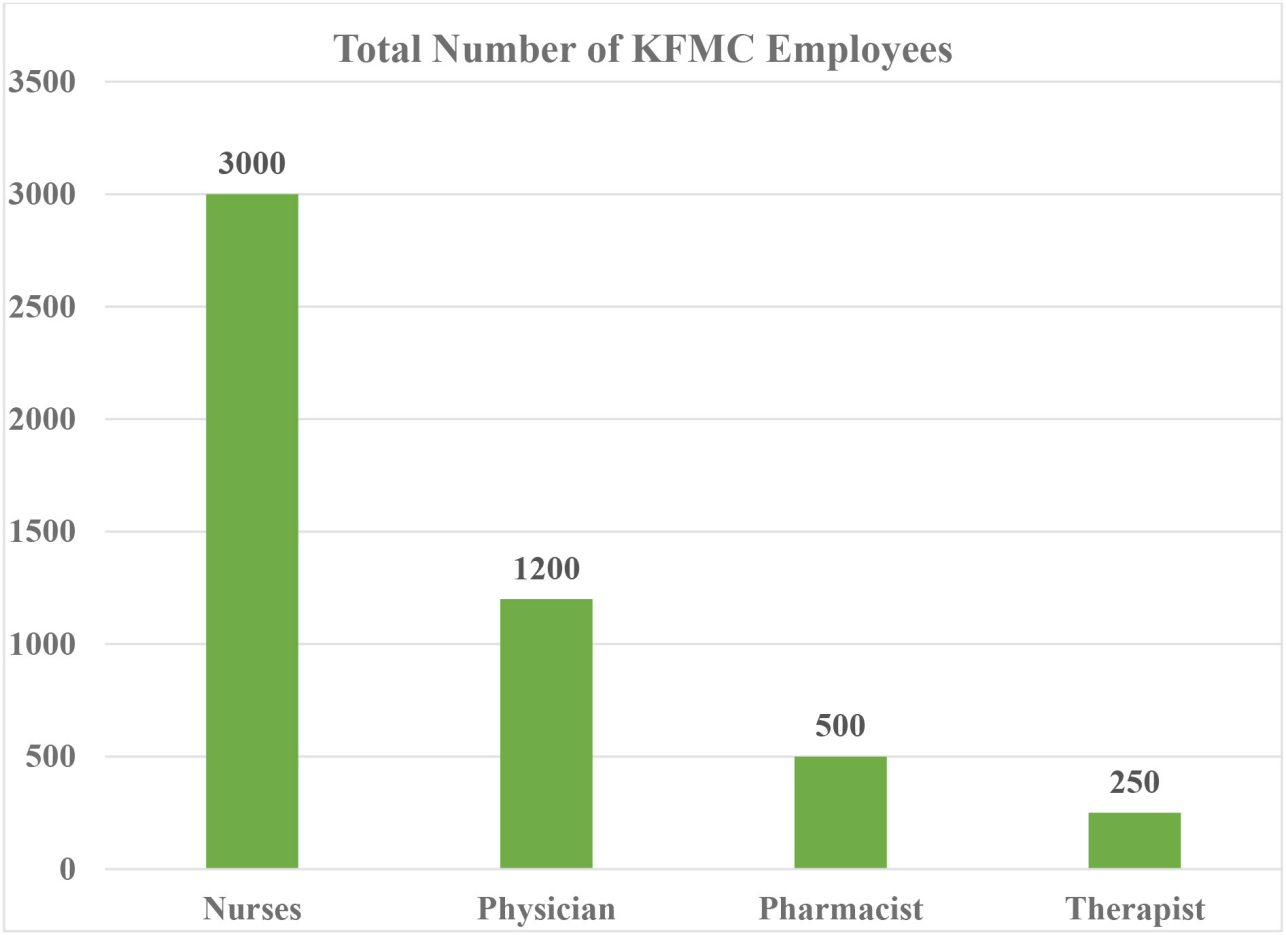
Total population of KFMC employee.

This study is a cross-sectional study conducted at King Fahad Medical City (KFMC), Riyadh, Saudi Arabia between June 2019 and February 2020 (Figure 1). KFMC has a total of 5,000 employees, among whom 68% are women while 32% are men. The estimation of the sample was determined by PASS ^®^ software version 11.0.10 [1983-2011.NCSS, LLC]. A total of 385 eligible participants recruited from nursing, pharmacy, clinics and rehabilitation department. By considering the total population of KFMC and using population portion sampling technique with 80% power of test to detect significant difference among groups followed by 95% C.I and 5% margin of error.

The size of a sample varies depending on the requirements of each study, in case of this research groups of employees from KFMC answered a survey questionnaire to provide a comprehensive insight on the concept of telecommunication. A survey questionnaire was distributed among a sample of 385 health care professionals, and the response rate obtained was 96.10%. Therefore, a total of 370 participants enrolled in the study that provided sufficient information by adopting the approach of meaning saturation i.e., it was assumed that majority of the healthcare providers in the same hospital had a homogeneous viewpoint with reference to their knowledge and attitude toward telemedicine technology. The saturation approach will hence validate the data preciseness for a purposive sample. The purpose and design of the study were explained to these potential participants initially, and a written consent was obtained from each. Once the researcher had the consent of these participants, a survey was distributed and the responses were gathered to interpret the study results (12).

### 2.2. Recruitment of participants

The readings of the study are specific to the population of Saudi Arabia. A structured self-administered questionnaire was used to record the responses of 370 healthcare providers from KFMC. The participants included in the study were physicians, nurses, physical therapists, and occupational therapists. All other health care professionals absent during the study, and not willing to participate were excluded from the study. The purpose and problem statement of the study were explained to the potential participants, and after getting their signed consent the questionnaire was distributed in their respective department according to population proportion sampling technique. Therefore, step by step every questionnaire was filled by participants and was double-checked by the researcher to identify any question missed or a non-responsive participant.

In order to control the parasitic variables of the study, the researcher will match participants in the telemedicine and control groups on key demographic or clinical characteristics which will help control for variables that might influence the results of the study. Besides this, establishing standardized protocols for the implementation of telemedicine services will further help control for parasitic variables that might influence the results of the study.

### 2.3. Data collection instrument

Data was collected using a structured self-administered questionnaire as a data acquisition tool that was adapted from previous studies and existing literature on telemedicine (13). The data collection tool, i.e., a survey questionnaire, comprised of three parts; demographic data, assessing the knowledge of healthcare professionals on telemedicine and evaluating the attitude of healthcare professionals toward telemedicine.

The first part included the demographic data related to the participants (7 items), such as gender, age, nationality, education, profession, work experience, and monthly income. The second part (9 items) was designed to assess healthcare professionals' knowledge of telemedicine by determining the participant's awareness and training on telemedicine, telemedicine technology, healthcare quality, effects of telemedicine, and benefits of telemedicine. The level of participants' knowledge regarding telemedicine was determined by their responses “Yes” or “No,” each “Yes” response was awarded a score of “1” while each “No” was given a score of “0.” In this section a participant could achieve a maximum score of 9 for the knowledge component and a minimum score of 0. The total scores were classified as poor (<50%), good (50–70%), and excellent (>70%). The third part investigated the healthcare professionals' attitudes toward telemedicine. This section consisted of 24 items rated on a 5-point scale ranging from 1 (strongly agree) to 5 (strongly disagree). These 24 items referred to the following perceived telemedicine attributes: relative advantage, compatibility, complexity, trial ability and observability. The scores for each of the statement in the third section were averaged to evaluate a specific mean value. A mean score of 2.5 (50%) was considered as poor attitude, 2.6 (51%)−3.0 (59%) as moderate attitude, and above 3.0 (60%) was marked as good attitude of healthcare professionals toward telemedicine technology.

### 2.4. Defining study domains

The perceived attributes of telemedicine technology examined in this study to assess the attitudes of healthcare professionals toward the new advancement include the following:

- Relative advantage:
  Relative advantage is defined as a degree to which a new product, technology, innovation or service is perceived as superior to the already existing idea. Relative advantage basically determines the rate of adoption of a new technology or service.
- Compatibility:
  Compatibility is referred as the ability to work or perform together due to well-matched and homogeneous characteristics or approach. Compatibility also verifies the consistency of an idea and the fact of being in agreement to it.
- Complexity:
  Complexity is defined as the quality of a phenomenon or idea to be complicated, confusing or intricate. Complexity is also referred as a condition where a phenomenon is difficult to understand or adopt.
- Trial ability:
  Trial ability defines that how easy and convenient it is for potential users to adopt and investigate a new product or technology. It facilitates the successful adoption of new innovations by enabling users to test run a technology or product before its implementation.
- Observability:
  Observability is referred as the ability to deduce and infer the internal states and operations of a system by analyzing its output and consequences. It is a process of management strategy that allows adopters of an innovation to focus on the most critical and relevant issues in a system.

### 2.5. Statistical analysis procedure

Data gathered in the study was presented as numbers, percentages, and Mean ± S.D. Parametric tests were used to compare groups on normally distributed variables and the normality of data was confirmed by the Kolmogorov-Smirnov test. Chi-square and Fisher's exact test were also incorporated according to whether or not the cell expected frequency was smaller than 5 and applied to determine the significant association between categorical variables. ANOVA *t*-test was applied to determine the mean significant differences between the dimension score (attitude of telemedicine) and profession. A stepwise regression test was used to determine the significant factors and predictors impacting the attitude toward telemedicine. A two-tailed (*P* < 0.05) was considered statistically significant. All data was entered and analyzed through the Statistical Package for the Social Sciences (SPSS 25), (SPSS Inc., Chicago, IL, USA).

### 2.6. Ethical considerations

The study was approved by the Institutional Review Board of the King Fahad Medical City Hospital. Consent was obtained from all subjects after explaining them the purpose of the study. Data was only collected from participants who gave a signed consent. Participation in the study was purely voluntary. Confidentiality and anonymity were maintained throughout the study as the participant's identity was not revealed in any stage of the research.

## 3. Results

Out of 385 distributed questionnaires, 370 participants were enrolled in the study, provided that the response rate was 96.10%. Of all participants, 112 (30.3%) were male and the remaining 258 respondents (69.7%) were female. Regarding the age group, more than half of the participants, i.e., 194 (52.2%), belonged to the age group of 30–40 years. The majority of participants i.e., 283 (76.7%) had a bachelor's degree. The distribution of the health care professionals was as follows: 90 (24.6%) were physicians, 230 (62.8%) were nurses and 46 (12.6%) were other allied health care professionals. The average score of telemedicine's knowledge was 11.12 among all participants. The demographic characteristics of the participants are represented clearly in Table 1.

**Table 1:** Basic demographic characteristics of participants (*n* = 370).

**Characteristics**	**Description**	***n* (*n*%)**
Gender	Male	112 (30.3%)
	Female	258 (69.7%)
Age group	<30	99 (26.6%)
	30–40	194 (52.2%)
	>40	79 (21.2%)
Nationality	Saudi	134 (36.1%)
	Non-Saudi	237 (63.9%)
Education	Diploma	38 (10.3%)
	Bachelors	283 (76.7%)
	Masters	44 (11.9%)
	PhD	4 (1.1%)
Type of profession	Physician	90 (24.6%)
	Nurse	230 (62.8%)
	Allied health care	46 (12.6%)
Working experience (years)	<5	98 (26.3%)
	5–10	141 (37.9%)
	>10	133 (35.8%)
Monthly income (SAR)	<5,000	61 (16.4%)
	5,000–10,000	181 (48.7%)
	>10,000	130 (34.9%)
Knowledge score of telemedicine	Median [IQR]	11.12 [77.78–1.12]

IQR, Interquartile Range; PhD, Doctor of Philosophy; SAR, Saudi Arabian Riyal.

The knowledge scoring of telemedicine was classified as poor (<50%), good (50–70%) and excellent (>70%). Two hundred and thirty-seven (63.7%) participants showed poor knowledge of telemedicine, 41 (11%) participants showed good knowledge, and only 94 (25.3%) participants exhibited excellent knowledge. There was a statistically significant (*P* < 0.001) association observed between the age, gender, education, nationality, profession, and the knowledge score of telemedicine (Table 2).

**Table 2:** Impact and association between knowledge score of telemedicine and study factors.

		**Knowledge score of telemedicine**			***P*-value**
		**Poor**	**Good**	**Excellent**	
Gender	Male	48 (20.3%)	15 (38.5%)	49 (52.1%)	<0.001
	Female	189 (79.7%)	24 (61.5%)	45 (47.9%)
Age group	<30	71 (30.0%)	15 (36.6%)	13 (13.8%)	0.001
	30–40	128 (54.0%)	17 (41.5%)	49 (52.1%)
	>40	38 (16.0%)	9 (22.0%)	32 (34.0%)
Nationality	Saudi	71 (30.1%)	17 (41.5%)	46 (48.9%)	0.004
	Non-Saudi	165 (69.9%)	24 (58.5%)	48 (51.1%)
Education	Diploma	31 (13.2%)	5 (12.2%)	2 (2.1%)	<0.001
	Bachelors	194 (82.9%)	33 (80.5%)	56 (59.6%)
	Masters	8 (3.4%)	3 (7.3%)	33 (35.1%)
	PhD	1 (0.4%)	0 (0.0%)	3 (3.2%)
Type of profession	Physician	30 (12.8%)	12 (30.8%)	48 (52.2%)	<0.001
	Nurse	175 (74.5%)	21 (53.8%)	34 (37.0%)
	Allied health care	30 (12.8%)	6 (15.4%)	10 (10.9%)
Work experience (years)	<5	74 (31.2%)	13 (31.7%)	11 (11.7%)	0.007
	5–10	85 (35.9%)	14 (34.1%)	42 (44.7%)
	>10	78 (32.9%)	14 (34.1%)	41 (43.6%)

PhD, Doctor of Philosophy.

The total mean score of the overall attitudes of health care professionals was (3.26 ± 0.51), which reflects a good attitude toward telemedicine among the healthcare professionals. The mean scores of attitudes dimensions toward telemedicine among the different types of professions were also computed independently. The overall mean score of the attitudes of physicians was (3.69 ± 0.54, good), which was higher than other health care professionals. The overall mean score of the attitudes of nurses was also good except for the following two dimensions; complexity (2.99 ± 0.47) and observability (2.79 ± 0.81), which showed a moderate attitude toward telemedicine.

As a result, the health care professionals depicted a good attitude in all dimensions toward telemedicine. This can be interpreted from the Mean ± SD values of the study domains; relative advantage (3.47 ± 0.76), compatibility (3.30 ± 0.80), complexity (3.14 ± 0.63), trial ability (3.43 ± 0.78) and observability (3.15 ± 1.08). Table 3 is a visual representation of these results.

**Table 3:** Comparative analysis of dimension scores among health care professionals.

	**Total**	**Physician**	**Nurse**	**Allied health care**	***P*-value**
Relative advantage	3.47 ± 0.76	4.07 ± 0.78	3.22 ± 0.61	3.58 ± 0.79	<0.001
Compatibility	3.30 ± 0.80	3.87 ± 0.91	3.06 ± 0.62	3.46 ± 0.8	<0.001
Complexity	3.14 ± 0.63	3.55 ± 0.8	2.99 ± 0.47	3.13 ± 0.64	<0.001
Trial ability	3.43 ± 0.78	4.07 ± 0.83	3.18 ± 0.62	3.46 ± 0.73	<0.001
Observability	3.15 ± 1.08	4.07 ± 1.09	2.79 ± 0.81	3.15 ± 1.17	<0.001
Overall attitude score	3.26 ± 0.51	3.69 ± 0.54	3.07 ± 0.37	3.31 ± 0.50	<0.001

Table 4 represents the regression model, where attitudes scores were the dependent variables. The model's R^2^ represents the variance in the attitudes toward telemedicine i.e., the impact of education explained 12.4% of the variance in the attitude toward telemedicine. Also, the impact of nationality explained 4.7% of the variance in the attitude toward telemedicine (for model 2). Furthermore, in models 3 and 4, the impact of age groups and work experience explained 1.6 and 1.00% of the variance in the attitude toward telemedicine, respectively. In model 5, the impact of gender explained 6.8% of the variance in the attitude toward telemedicine.

**Table 4:** Identification of factors affecting the attitude of telemedicine by regression analysis.

**Attitude scores**		**Unstandardized coefficients**		**Standardized coefficients**	**t**	**R^2^**	***P*-value**
**Model**		**B**	**Std. error**	β			
Step 1	Education	0.256	0.051	0.256	4.986	0.124	<0.001
Step 2	Nationality	−0.271	0.065	−0.254	−4.183	0.047	<0.001
Step 3	Age group	0.101	0.052	0.136	1.931	0.016	0.054
Step 4	Work experience (years)	0.068	0.048	0.105	1.423	0.005	0.156
Step 5	Gender	−0.099	0.06	−0.089	−1.649	0.068	0.100

## 4. Discussion

The successful implementation of any technology is predominantly influenced by a number of factors. Human-related factors are of great significance, especially when implementing any technology in the healthcare system. This proposed study basically explored the knowledge and attitude of healthcare professionals toward telemedicine by conducting a survey among healthcare professionals at KFMC. The results show that healthcare professionals play a vital role in the practical implementation of telemedicine and the sustainability of health care technology. Therefore, adequate knowledge of the new innovation and a good attitude of medical specialists toward such a technology are mandatory so that telemedicine can be implemented extensively (13).

Literature includes many relevant studies on the role and significance of telemedicine in the medical industry. Telemedicine approach helps to reduce the costs and minimize the traveling of patients's from remote areas to metropolitan cities (14, 15). According to a recent Chinese research, <5% of research respondents were of the viewpoint that telemedicine doesn't play a helpful role in improving the healthcare system and mitigating the medical cost and burden on patients whereas the majority were in favor of the new medical advancement (16). Moreover, the literature also reveals that using teleconsultation and by receiving distant medical education, the new and grassroots doctors can learn and gain guidance on the treatment plans by communicating with senior medical experts in larger cities which will eventually enhance their medical skill (17). A group of Chinese researchers claimed that majority of the medical staff and professionals were willing to adopt and promote the telemedicine technology in China (16). Similar results were generated by a survey study conducted in Australia where around 61.9% of participants revealed that they had a “better” experience with adopting telemedicine rather than using the traditional appointment scheduling medical system (18).

Adding further, a study of Saudi Arabia propounded that majority of the medical professionals readily accepted the implementation of telemedicine in hospitals (15). A German survey demonstrated that maximum number of postgraduate medical officers are satisfied by the implementation of telemedicine and are willing to get more training (19). According to a US study, international oncologists revealed that 73.8% of them were satisfied by the adoption of telemedicine and after the outbreak of COVID-19 this percentage escalated to 81.5% (20). Moreover, the ESAIC (European Society of Anaesthesiology and Intensive Care) and ASA (American society of Anaesthesiology) collaborated to conduct a survey which claimed that anaesthesiologists are highly satisfied by the use of telemedicine technology and around 86.3% of them are willing to pursue the use of telemedicine in their clinical practices (21).

Additionally, the survey conducted by Michigan State University in the US and other relevant studies manifest that the attitude of healthcare professionals and their viewpoint regarding telemedicine usage are key components to figure out the successful implementation of this technology (22, 23). To deal with human-related constraints in the development of a new technology like telemedicine, countries and medical industries need to frame targeted strategies. The purpose of this study is to assess the knowledge level and attitude of healthcare professionals at KFMC regarding the implementation of telemedicine in Saudi Arabia. The study results interpreted that 63.7% of the total 370 respondents had poor knowledge regarding telemedicine, which is still less than the percentage of European healthcare professionals, i.e., 84%, who have little awareness on telemedicine (24). However, the mean score of the participants was >3.0 which shows that on a whole the medical professionals at KFMC have a good attitude toward the implementation of telemedicine technology. Besides this, the study model suggested that education had a 12.4% impact on the variance in the attitude toward telemedicine which suggests that efforts need to be put forward in order to educate healthcare professionals and increase their awareness and training on telemedicine for the sustainable adoption of this advancement in Saudi Arabia.

In comparison to the findings of this study, a research was conducted in the teaching hospitals in Mashhad, Iran which showed that although most of the research participants had a positive attitude toward telemedicine (65%) but their knowledge on telemedicine was signified to be at a lower level with a mean score of 13 ± 5.5 (23). These results are hence similar to the findings of this proposed research as both indicate poor knowledge of healthcare professionals on telemedicine and a positive attitude toward the adoption of this new technology. Another study conducted in Isfahan, Iran reveals that 63.3% of its participants have a good attitude toward telemedicine's implementation hence complying with the findings of this research (25). Therefore, the findings of this research clearly indicate that the knowledge level of most healthcare professionals is poor but their attitude toward the adoption of telemedicine is positive which acknowledges other literature findings from similar studies.

As telemedicine is a new development in the field of medical services, its implementation has still not been satisfactory and far-reaching. However, the favorable attitude of healthcare professionals in Saudi Arabia ensures their ability to offer Continuing Medical Education (CME) for ground roots doctorsin rural and remote areas in addition to providing remote consultations to patients from expert medical specialists (26). The research findings also signify the need to enhance the knowledge and skills of health care professionals by introducing targeted interventions and training programs before the complete implementation of telemedicine. Thus, these interpretations provide a great opportunity for implementing telemedicine fully in the medical system of Saudi Arabia, provided that adequate training and education sessions are provided to the healthcare professionals. Hence before the extensive implementation of this technology, it is crucial to increase the user knowledge level of telemedicine and make them aware of the competencies and advantages of this advancement. This will validate the successful use of telemedicine technology in the coming future and promote sustainability in the medical infrastructure.

## 5. Challenges and future recommendations

The challenges that impede the wide-scale adoption of telemedicine include inadequate medical infrastructure, such as a lack of necessary equipment, hardware, and software, as well as unreliable internet connections. Additionally, there are costs associated with updating to telemedicine technology. One of the biggest challenges is raising concerns regarding training, awareness and acceptance of telemedicine among healthcare professionals and the general public. Furthermore, a lack of education is a significant obstacle to the implementation of telemedicine in many developing countries (27).

Thus, there is a need to adapt policies that increase the awareness regarding telemedicine applications and its benefits among the laymen, makes telemedicine knowledge and training more accessible to medical staff at hospitals and universalize telemedicine in all remote and under developed areas through media coverage and training programs. Providing incentives to medical staff on adopting telemedicine in their system will enhance their satisfaction, utilization and willingness to continue the usage of this advancement in clinical practices. The increased convenience and cost reduction following telemedicine adoption will further raise the satisfaction level of healthcare professionals. Cost subsidy incentives, expense strategies and professional title promotions must also be offered in hospitals to promote the successful implementation and popularity of telemedicine.

To overcome the challenges associated with implementing telemedicine in Saudi Arabia, a number of different types of awareness and training sessions will likely be needed. Some key areas that will likely need to be addressed include:

- Technical training: Medical staff will need to be trained on the technical aspects of telemedicine, including how to use the necessary equipment, hardware, and software.
- Clinical training: Medical staff will also need to be trained on the clinical aspects of telemedicine, including how to conduct virtual consultations, remote monitoring, and other telemedicine-related procedures.
- Legal and regulatory training: Medical staff will need to be aware of the legal and regulatory requirements related to telemedicine in Saudi Arabia, including issues related to data privacy and security, patient consent, and reimbursement.
- Communication training: Medical staff will also need to be trained on how to effectively communicate with patients during virtual consultations, as the physical presence of the doctor is not there, which can be a barrier in trust factor between the doctor and the patient.
- Public awareness campaigns: The general public will need to be educated about the benefits of telemedicine and how to access telemedicine services.
- Incentives for adoption: To encourage healthcare professionals to adopt telemedicine, various incentives such as cost subsidies, expense strategies, and professional title promotions should be offered.

## 6. Strengths and limitations

Telemedicine has both advantages and disadvantages. One limitation is that it can't provide the same level of detail as an in-person examination. Additionally, the use of a screen can make some patients feel less comfortable, which can make it harder to build trust with their doctor. Some patients may also have difficulty communicating their health concerns through online consultations. However, telemedicine also has several strengths that should not be overlooked. For example, it can be very effective in controlling the spread of infectious diseases such as COVID-19. It is also more convenient and can reduce the need for unnecessary emergency room visits. Additionally, it can be more cost-effective by saving on travel expenses for routine check-ups. Telemedicine can also provide access to healthcare in rural areas with limited medical facilities. Additionally, online therapy-led support groups can provide peer-to-peer support for people dealing with similar challenges. Overall, telemedicine has its pros and cons when applied on a larger scale.

The major limitation of this study was that the participants were recruited from a single hospital (KFMC) due to the limited resources of the researchers. As a result, the study findings were confined to a smaller medical population affecting the credibility of the results. The study should have been conducted using a larger sample of healthcare professionals from all across the country to evaluate the knowledge level and attitude of healthcare professionals more precisely. This would have also increased the generalizability of the research findings. Moreover, this study analyses the telemedicine approach from the perspective of Saudi healthcare professionals only, neglecting the viewpoint of patients. The scope of this study can be expanded further by combing and comparing the perspectives of both patients and medical professionals simultaneously in a single research. Analyzing the impact of COVID-19 on the usage of telemedicine technology and the attitude of healthcare professionals can also be studied in future research to provide an in-depth perspicacity on the topic of telemedicine. Lastly, this study was based on a self-administered survey questionnaire which can increase the chances of researcher bias and impact the output of the study.

Considering the strengths, this study provides a multi-level understanding of telemedicine technology by discussing the experience, knowledge level and attitude of healthcare professionals, and the challenges and expectations from the development and implementation of this advanced technology. It is also one of the few studies using regression models to analyse the factors that impact healthcare professional's attitude and satisfaction level toward telemedicine.

## 7. Conclusion

This study declares that healthcare professionals are the key users of telemedicine technology and their perceptions and knowledge on telemedicine plays a crucial role in the sustainable development of health industry. The use of telemedicine in Saudi Arabia is insufficient and limited which is why many healthcare professionals have low-grade knowledge about the new medical concept, only 25.3% of participants were having good knowledge of telemedicine. However, the positive attitude of majority participants toward implementing telemedicine increases the scope of this technology in achieving a sustainable medical system. Variations in attitude exist among the different groups of healthcare professionals. Therefore, there is a need to establish tailored educational interventions for healthcare professionals to ensure the proper implementation and sustainability of telemedicine. To conclude, as the knowledge deficit can have an impact on the success of the implementation of telemedicine, it is vital to build targeted interventions to train the health care professionals in telemedicine, which would aid in ensuring the proper implementation and sustainability of this technology. Telemedicine technology is thus a salvation to the medical industry and healthcare systems, opening a gateway to improved medical services for patients all across the globe.

Telemedicine technology is on the verge of revolutionizing healthcare, and it's not too far in the future when most of our healthcare-related issues will be addressed virtually. With only a little further development and improvement, telemedicine technology has the potential to benefit the entire world. Some of its research is already showing promising long-term results. As more people will be cared for without having to crowd hospitals or institutions, the technology will also make it possible for patients who require urgent physical encounters with their clinicians to receive treatment immediately. Introducing new technologies is never easy, and telemedicine has and will continue to face criticism and resistance, but over time it will become widely accepted and successfully used throughout the world.

## Data availability statement

The original contributions presented in the study are included in the article/supplementary material, further inquiries can be directed to the corresponding author.

## Ethics statement

Ethics review and approval/written informed consent was not required as per local legislation and institutional requirements.

## Author contributions

MB and DL contributed to the conception and design of the study. DL organized the database. MB performed the statistical analysis and wrote the first draft of the manuscript. SA and AA-S wrote sections of the manuscript. All authors contributed to manuscript's revision, read, and approved the submitted version.
